# Overexpression, Purification and Characterisation of the *Plasmodium falciparum* Hsp70-z (PfHsp70-z) Protein

**DOI:** 10.1371/journal.pone.0129445

**Published:** 2015-06-17

**Authors:** Tawanda Zininga, Ikechukwu Achilonu, Heinrich Hoppe, Earl Prinsloo, Heini W. Dirr, Addmore Shonhai

**Affiliations:** 1 Department of Biochemistry, School of Mathematical & Natural Sciences, University of Venda, Thohoyandou, 0950, South Africa; 2 Protein Structure-Function Research Unit, School of Molecular & Cell Biology, University of the Witwatersrand, Johannesburg 2050, South Africa; 3 Department of Biochemistry, Microbiology & Biotechnology, Rhodes University, Grahamstown 6140, South Africa; 4 Biotechnology Innovation Centre, Rhodes University, Grahamstown 6140, South Africa; Université Pierre et Marie Curie, FRANCE

## Abstract

Six Hsp70-like genes are represented on the genome of *Plasmodium falciparum*. Of these two occur in the cytosol: *P*. *falciparum* Hsp70-z (PfHsp70-z) and PfHsp70-1. PfHsp70-1 is a well characterised canonical Hsp70 that facilitates protein quality control and is crucial for the development of malaria parasites. There is very little known about PfHsp70-z. However, PfHsp70-z is known to be essential and is implicated in suppressing aggregation of asparagine-rich proteins of *P*. *falciparum*. In addition, its expression at the clinical stage of malaria correlates with disease prognosis. Based on structural evidence PfHsp70-z belongs to the Hsp110 family of proteins. Since Hsp110 proteins have been described as nucleotide exchange factors (NEFs) of their canonical Hsp70 counterparts, it has been speculated that PfHsp70-z may serve as a NEF of PfHsp70-1. In the current study, *P*. *falciparum* cells cultured *in vitro* were subjected to heat stress, triggering the enhanced expression of PfHsp70-z. Biochemical assays conducted using recombinant PfHsp70-z protein demonstrated that the protein is heat stable and possesses ATPase activity. Furthermore, we observed that PfHsp70-z is capable of self-association. The structural-functional features of PfHsp70-z provide further evidence for its role as a chaperone and possible nucleotide exchange factor of PfHsp70-1.

## Introduction

An understanding of the biology of the deadly malaria parasite, *Plasmodium falciparum*, is essential for the development of new therapeutic options in light of the increasing incidences of antimalarial drug resistance. The role of molecular chaperones, amongst them, heat shock proteins (Hsps) in the development of malaria parasites has been well documented [1][2][3]. Hsps play an important house-keeping role in the cell by facilitating protein folding and maintaining quality control [4][5]. The upregulated expression of some Hsps, has been reported to be crucial for the development of malaria parasites in the host [6][7]. It has also been suggested that the expression of certain parasite Hsps is associated with clinical progression of the disease [8]. In addition, some Hsps are essential for parasite viability, while others have been proposed as potential antimalarial drug targets [1][3][6][7].

Hsp70s are implicated in protein degradation, protein translocation and assembly and disassembly of oligomeric complexes [9][10][11]. Hsp70 chaperones are composed of an N-terminal nucleotide binding domain (NBD), which confers them with ATPase activity; and a C-terminal substrate binding domain (SBD). The two domains are connected by a highly conserved linker segment [12]. Hsp70s are divided into three sub-families: DnaK-like (canonical Hsp70s); Hsp110 and Grp170 [13]. DnaK is the prokaryotic form of Hsp70. DnaK/Hsp70 is capable of refolding misfolded protein and suppressing protein aggregation [14]. DnaK and other Hsp70s that closely resemble DnaK are considered to be canonical Hsp70s. On the other hand, Hsp110 members are specialised proteins that are structurally distinct from canonical Hsp70s. They have been described as nucleotide exchange factors (NEFs) of canonical Hsp70s [15]. Hsp110 and Grp170 members are closely related but whereas Hsp110 occurs in the nucleus and cytosol, the Grp170 is characterised by endoplasmic reticulum signals which restrict its localisation to this cellular compartment [13]. Hsp110 and canonical Hsp70s both possess a highly conserved NBD and a less well conserved C-terminal SBD. Hsp110 differs from canonical Hsp70s as the former possess an extended lid segment [16]. ADP-bound Hsp70s bind to their substrates with high affinity and release them in their ATP-bound states [17]. The cycle between ADP- and ATP-bound states is regulated by nucleotide exchange factors.

The *P*. *falciparum* genome encodes for 6 Hsp70 proteins [2]. The proteins occur in various cellular compartments: PfHsp70-1 (PF3D7_0818900) and PfHsp70-z (PF3D7_0708800) (cytosol); PfHsp70-2/PfBiP (PF3D7_0917900) and PfHsp70-y (MAL13P1.540) (endoplasmic reticulum); PfHsp70-3 (PF3D7_113400) (mitochondrion) and PfHsp70-x (PF3D7_0831700) which is exported to the erythrocyte cytosol; reviewed in [2]. Of particular interest to this study is PfHsp70-z which is predicted to function as the NEF of its cytosolic counterpart, PfHsp70-1 [2].

Hsp110s are known to inhibit protein aggregation by serving as substrate holdases [18]. For a long time, the role of Hsp110 was poorly understood until it was reported that yeast Hsp110 (Sse1p) and human Hsp110 (hHsp110) serve as NEFs of their respective canonical Hsp70 counterparts [19]. The NEFs of Hsp70 from prokaryotes and eukaryotes are structural distinct but they share a common role. GroP-like gene E (GrpE) is a NEF which occurs in prokaryotes and the mitochondria [20]. Eukaryotes employ various NEFs amongst them, Bcl2-associated athanagene-1 (Bag-1) [21]; heat shock protein binding protein 1 (HspBP1) [22]; and Hsp110 [15]. In *P*. *falciparum*, a GrpE homologue occurs in the mitochondrion [23] and none is resident in the cytosol. A *P*. *falciparum* cytosolic Bag-1 homology has not been identified. Therefore *P*. *falciparum* potentially has only one NEF in PfHsp70-z [24].

Although PfHsp70-z is thought to play an essential role through suppressing aggregation of malarial asparagine-rich proteins [25], it is possible that its function as NEF of Hsp70 may be crucial for parasite survival [24]. Nucleotide exchange indirectly determines the substrate dwell time on Hsp70 SBD [26]. Premature release of substrates from Hsp70 could result in their aggregation, leading to their degradation [10] and hence the role of NEFs is an important aspect of Hsp70 function.

Chloroquine resistance in *P*. *falciparum* is mapped to a 36 kb segment of Chromosome 7, which includes the *PfHsp70-z* (*PfCg4)* gene [27]. It is therefore important to characterise the role of PfHsp70-z towards understanding its broader functions. For example, Hsp110-Hsp70 complexes have been implicated in cell division where they modulate the activity of the molecules kenesin-5 motor and cin8, which are required for spindle elongation in yeast and mammalian cells [28]. The modulation of spindle elongation by molecular chaperones might be a mechanism in which cell division may be controlled under proteostatic stress. The rapid growth cycles of *P*. *falciparum* in the host facilitates its survival and may influence its pathogenesis [29]. Although it is unknown whether PfHsp70-z modulates spindle elongation in *P*. *falciparum*, it is important to fully characterise its role in the survival and development of malaria parasites.

In the current study, we expressed and purified recombinant PfHsp70-z protein and investigated its biophysical characteristics. We also investigated its expression in the malaria parasite subjected to heat stress. Our findings suggest that PfHsp70-z is a heat-inducible and heat stable molecule with ATPase activity that is capable of forming higher order oligomers. We discuss our findings and their implications on our understanding of PfHsp70-z in the development of *P*. *falciparum*.

## Material and Methods

### Materials

Unless, specified chemical reagents used in this study were purchased from Merck Chemicals (Darmstadt, Germany), Thermo Scientific (Illinois, USA), Zymo Research (USA), Melford (Suffolk, UK), Sigma-Aldrich (USA). Nickel NTA resin was purchased from Thermo Scientific (USA). ECL was bought from (Thermo Scientific, USA). α-His antibodies were purchased from (Sigma Aldrich, USA). The α-PfHsp70-z antibody produced in rabbit against peptide: CSLQEQEKNKPLYEP, corresponding to amino acids 768–782 of the PfHsp70-z amino acid sequence was secured from GenScript (USA). Polyclonal rabbit raised antibodies specific for PfHsp70-1 that were previously described [30] were used to validate the authenticity of recombinant PfHsp70-1 protein.

### Construction of expression vector pQE30-PfHsp70-z

A codon harmonized form of the *PfHsp70-z* gene (PlasmoDB accession number: P3D7_088000) was cloned into with pQE30 (Qiagen) vector using *Bam*HI and *Hind*III restriction. The construct was produced by GenScript (USA). The integrity of the pQE30-PfHsp70-z which was used to express N-terminal His-tagged PfHsp70-z was verified by restriction analysis and DNA sequencing.

### Overexpression and purification of PfHsp70-z

*E*. *coli* JM109 cells (ThermoFisher Scientific, USA) that had been transformed with the pQE30/PfHsp70-z construct were cultured at 30°C and then diluted 1 in 10. At mid-log phase (A_600_−0.6), PfHsp70-z expression was induced using 1 mM isopropyl-β-D-1-thiogalactopyranoside (IPTG). After overnight incubation, the cells were harvested by centrifugation at 5000 g for 20 min and the pellet fraction was suspended in lysis buffer (100 mM Tris–HCl, pH 7.4, 300 mM NaCl, 10 mM imidazole, 1 mM EDTA, 1 mM PMSF and 1 mg/ml lysozyme) at 23°C for 1 hr and was subsequently frozen at -80°C. The cells suspended in lysis buffer were allowed to thaw on ice for 1 hr. The cells were sonicated to facilitate cell lysis. Nucleic acids were precipitated by addition of 0.1% (w/v) Poly(ethyleneimine) [PEI]. The cell lysate was then centrifuged at 5000 g for 20 min. The (His)_6_-PfHsp70-z protein was purified using a HisPur nickel-charged nitrilotriacetic acid (Ni-NTA) immobilised metal affinity chromatography column (IMAC) following the manufacturer’s instructions. The protein was extensively dialyzed overnight at 4°C against a storage buffer (10 mM Tris, pH 7.5, 150 mM NaCl, 0.8 mM DTT, 10% (v/v) glycerol). The protein yield was estimated using the Bradford assay (Sigma-Aldrich, USA) with BSA as the standard. The authenticity of PfHsp70-z was confirmed by SDS-PAGE analysis coupled with Western blot using both rabbit α-His (Sigma-Aldrich, USA), and rabbit polyclonal peptide α-PfHsp70-z antibodies (GenScript, USA). HRP-conjugated anti-rabbit IgG (1:2000 dilution) (Thermoscientific, USA) were used as secondary antibodies. To validate that the purified PfHsp70-z protein was not contaminated traces of *E*. *coli* Hsp70 (DnaK), we probed the protein preparations using α-DnaK antibodies (Stressgen, Germany) by Western blotting technique. Imaging of the protein bands on the blot was conducted using the ECL kit (Thermoscientific, USA) as per manufacturer’s instructions. Images we captured using ChemiDoc Imaging system (Bio-Rad, USA).

### Investigation of heat-induced expression of PfHsp70-z in *Plasmodium falciparum* 3D7 cells

*P*. *falciparum* 3D7 cells were cultured as previously described [1][31]. Sorbitol synchronised parasites were harvested at the trophozoite stage from two fractions. One fraction consisted of parasites that had been subjected to heat shock at 42°C for 2 hr and the other were cultured under normal temperature conditions (37°C) for 2 hr prior to harvesting. Parasites were collected by centrifugation at 2000 g for 10 min after saponin (0.1%), followed by lysis of erythrocytes. This was followed by an extensive wash step using PBS (pH 7.4). The parasite lysate was subjected to Western analysis to confirm the expression of PfHsp70-z using rabbit polyclonal peptide α-PfHsp70-z antibodies and HRP-conjugated anti-rabbit IgG secondary antibodies (1: 2000) (Thermoscientific, USA). To validate the specificity of the α-PfHsp70-z antibodies rabbit pre-immunization serum was also used. Glycophorin was also probed as a loading control using goat α-glycophorin (Sigma-Aldrich, USA) and HRP-conjugated anti-goat secondary antibodies (1: 5000) (Sigma-Aldrich, USA). Furthermore, we conducted Western blotting analysis using α-PfHsp70-z antibodies to confirm the specificity of the antibodies by probing their capability to bind recombinant PfHsp70-z and not recombinant PfHsp70-1 [30]. Images were acquired using ChemiDoc Imaging system (Bio-Rad, USA) and densitometric analysis of bands were conducted using the Image Lab version 5.1 built 8 (Bio-Rad, USA).

### Investigation of the secondary and tertiary structural organisation of PfHsp70-z

The secondary structure of recombinant PfHsp70-z was analysed using Far-UV circular dichroism (CD). CD spectroscopy experiments were performed using a J-1500 CD spectrometer (JASCO Ltd, UK) equipped with a Peltier temperature controller. PfHsp70-z recombinant protein (2 μM) was suspended in 20 mM Tris-HCl pH 7.4, 100 mM sodium fluoride buffer and analysed using a 0.1 cm path-length quartz cuvette (Hellma). Spectra were averaged for least 15 scans after subtraction of baseline scan (buffer in which PfHsp70-z protein was excluded). The CD measurements were normalised to protein concentration and presented as molar ellipticity, [θ] deg.cm^2^.dmol^-1^. Secondary structure predictions were conducted on the observed CD spectrum at constant temperature set at 19°C. The spectra were deconvulted to α-helix, β-sheet, β-turn and unordered regions, using the Dichroweb server, http://dichroweb.cryst.bbk.ac.uk [32][33].

We further investigated the effect of heat and urea on PfHsp70-z, as thermal and chemical denaturants, respectively. The secondary structure of the protein was monitored at 222 nm as the temperature was raised monotonically initially from 19°C to 65°C at a rate of 0.5°C per min. The temperature was restored to 19°C. The experiment was repeated, this time the temperature was raised from 19°C to 95°C at a rate of 0.5°C. The folded state of the PfHsp70-z protein at any given temperature was determined as follows [34]:

$$\left({\left[\theta \right]}^{\text{t}}-{\left[\theta \right]}^{\text{h}}\right)\;/\;\left({\left[\theta \right]}^{\text{l}}-{\left[\theta \right]}^{\text{h}}\right)$$ Equation 1

Where [θ]^t^ is the molar ellipticity at any given temperature, [θ]^h^ at highest temperature, and [θ]^l^ at lowest temperature, respectively.

The effects of urea on the stability of PfHsp70-z was monitored by assessing the molar residue ellipticity at 222 nm, with temperature set at 19°C in the absence and presence of varying urea concentration (1 M to 10 M). The folded state of PfHsp70-z protein exposed to various levels of urea was calculated using the Eq 1, described above by substituting the respective urea concentrations for the temperature values.

Next, we monitored changes in the tertiary structure of the protein that was subjected to urea denaturation by conducting fluorescence based analysis. The recombinant PfHsp70-z protein (0.45 μg/ml) was incubated in assay buffer A (25 mM HEPES-KOH pH 7.5, 100 mM KCl, 10 mM MgOAc) for 20 min at 20°C in the presence of the variable urea concentrations (1 M- 8 M). Fluorescence spectra were recorded between 300 and 400 nm after initial excitation at 295 nm using JASCO FP-6300 spectrofluorometer.

### Assessment of the capability of PfHsp70-z to form higher order oligomers

The capability of PfHsp70-z molecules to self-associate was conducted using a Proteon XPR36 (BioRad, USA) surface plasmon resonance (SPR) machine. The assays were conducted at room temperature (25°C), filter sterilised and degassed PBS-Tween (10 mM Phosphate, 137 mM NaCl, 3 mM KCl, 0.005% (v/v) Tween 20 and 20 mM EDTA; pH 7.4) was used as running buffer. PfHsp70-z (as ligand) was immobilised at concentrations of 0.5 μg/mL and 1 μg/mL. At these concentrations we achieved 187 response units (RU) for PfHsp70-z per immobilization surface [35]. The immobilisation of ligands was achieved through covalent attachment to the modified alginate polymer layer on the GLC sensor chip via amine coupling following a protocol that was provided by the manufacturer (BioRad, USA). As analytes, aliquots of PfHsp70-z and BSA (as negative control), were prepared at final concentration of 2000, 1000, 500, 250, and 125 nM and were injected into each horizontal channel at a rate of 50 μl/min. The analysis was conducted in the absence or presence of 5 mM of either ATP or ADP. Association was allowed for 2 min, dissociation was monitored for 10 min. The SPR sensorgrams were fit to a simple Langmuir kinetic binding model to calculate the association rate constant (*k*_*a*_ in the unit of M^-1^s^-1^), dissociation rate constant (*k*_*d*_ in the unit of s^-1^) and the equilibrium constant (*K*_*D*_ in the unit of M). The SPR data were analysed using ProteOn Manager software version 3.1.0.6. by concatenating the responses of all five analyte concentrations [36].

### Investigation of ATPase activity of PfHsp70-z

The ATPase activity of PfHsp70-z was determined by quantifying the amount of released inorganic phosphate based on a previously described approach [37]. Briefly, 0.4 μM PfHsp70-z was incubated for 5 min in buffer HKMD (10 mM HEPES-KOH pH 7.5, 100 mM KCl, 2 mM MgCl_2_, 0.5 mM DTT). The reaction was started by addition of ATP and samples were collected after 30 min. Kinetic plots for the ATPase activities of PfHsp70-z and PfHsp70-1 were determined by generating Michaelis–Menten plots. At least three independent batches of recombinant protein were used in separate experiments. Statistical analysis was conducted using GraphPad prism 6.05.

## Results

### Overexpression and purification of recombinant forms of PfHsp70-z and PfHsp70-1

**Fig 1:**
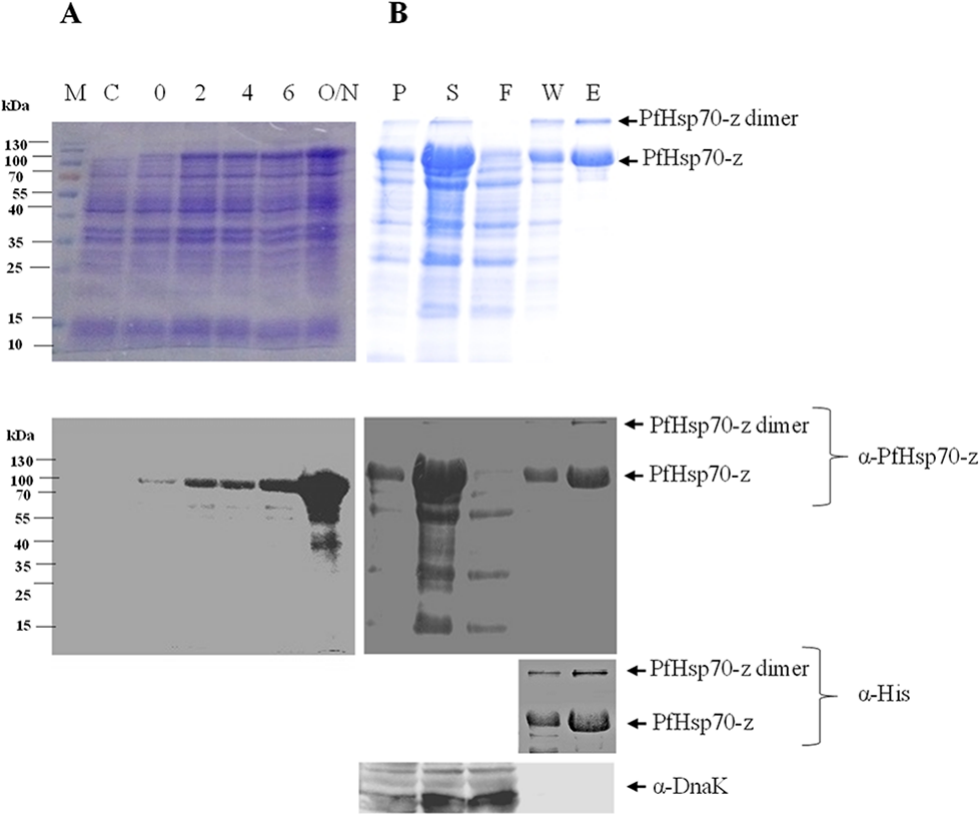
Expression and purification of recombinant PfHsp70-z. PfHsp70-z was expressed in *E*. *coli* JM109 cells transformed with pQE30/PfHsp70-z. SDS-PAGE (12%) and Western analyses of the expression (A) and purification (B) of recombinant PfHsp70-z; lane M–Page ruler (Thermo Scientific) in kDa; lane C—total extract for cells transformed with a neat pQE30 plasmid; lane 0 –total cell extract transformed with pQE30/PfHsp70-z prior to IPTG induction; lanes 2, 4, 6, O/N–total cell lysate obtained 2, 4, 6 and 16 h post induction, respectively. Lane P, S-pellet and soluble fractions obtained from the total lysate for cells transformed with pQE30/PfHsp70-z, respectively; lane F; flow through; lane W—wash sample, and lane E—PfHsp70-z protein eluted using 500 mM imidazole. *Lower panel*s: Western blot based on use of α-PfHsp70-z and α-(His)_6_ antibodies confirming expression and purification of PfHsp70-z protein. To confirm the purity of PfHsp70-z from DnaK contamination, Western blotting was conducted using α-DnaK antibodies.

**Fig 2:**
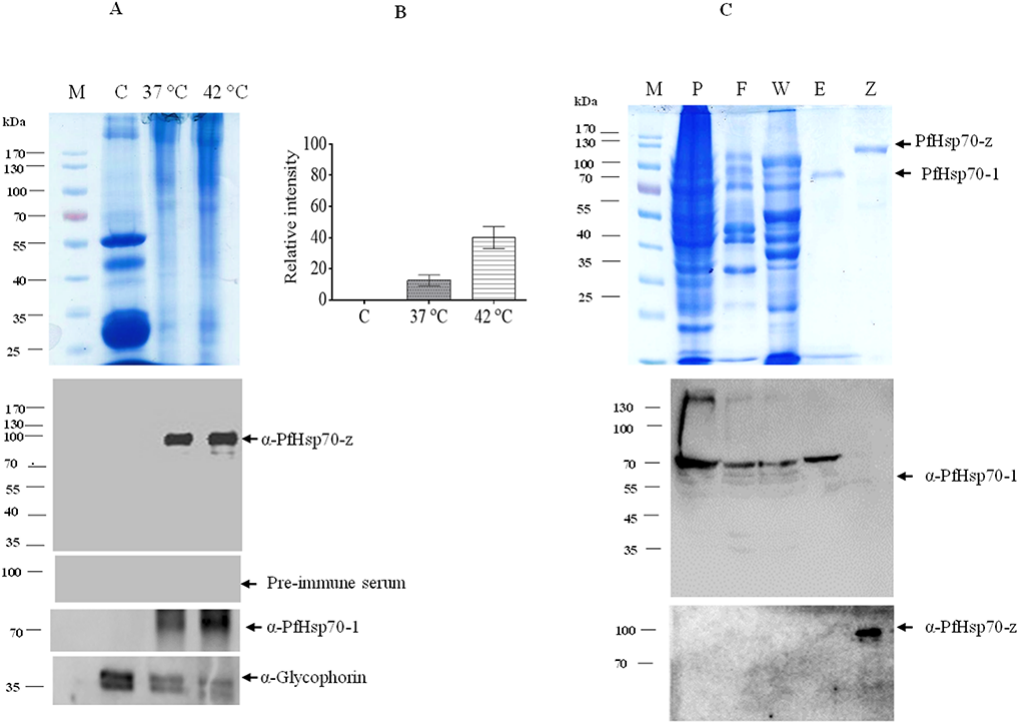
PfHsp70-z is induced by heat stress in *Plasmodium falciparum* parasites cultured at the blood stage. SDS-PAGE (12%) and Western analyses of the expression of PfHsp70-1 and PfHsp70-z by *P*. *falciparum* parasites cultured at 37°C and 42°C, respectively (panel A); lane M (molecular weight markers in kDa); lane C (uninfected erythrocyte cell lysate); and parasite lysate harvested from erythrocytes infected by *P*. *falciparum* parasites cultured in vitro at 37°C and 42°C, respectively; densitometric analysis validating the upregulated expression of PfHsp70-z in parasites cultured at 42°C compared to culture incubated at 37°C (panel B). The expression of PfHsp70-z in response to heat stress was validated by densitometric analysis. A t-test was used to validate the upregulated expression of the protein at 42°C compared to 37°C (*p<0*.*005*); SDS-PAGE and Western blot analyses for the expression and purification of recombinant PfHsp70-1 protein (panel C), lane P, S-pellet and soluble fractions, respectively, obtained from the total lysate for cells transformed with pQE30/PfHsp70-1, respectively; lane F—flow through; lane W—wash sample; lanes E—PfHsp70-1 protein eluted using 250 mM imidazole, and lane Z–recombinant PfHsp70-z protein. *Lower panel*s: Western blot analyses conducted using α-PfHsp70-1 and α-PfHsp70-z antibodies, respectively.

PfHsp70-z and PfHsp70-1 recombinant proteins were expressed and subsequently purified using nickel affinity chromatography (Figs 1 and 2). PfHsp70-z was eluted as a species of approximately 110 kDa (Fig 1B). However, we also observed a band running at approximately 200 kDa which was recognised by both α-His and α-PfHsp70-z antibodies. We surmised that this band possibly represented a dimer form of PfHsp70-z. The purity of the eluted protein was further validated by probing using α-DnaK antibodies and our assessment confirmed that the eluted PfHsp70-z protein was free from DnaK contamination (Fig 1B; *lower panel*). We conducted a Western blot on recombinant PfHsp70-1 using the α-PfHsp70-z antibodies which we developed and validated that they do not cross-react with PfHsp70-1 (Fig 2C). Therefore, the α-PfHsp70-z antibodies are able to distinguish between these two chaperones which are resident in the parasite cytosol.

### Expression of PfHsp70-z is upregulated in response to heat stress

The development of malaria parasites at the blood stage is associated with periodic fever conditions. These fever conditions are known to induce select heat shock proteins which are required for maintaining proteostasis in the parasite [8]. In addition, the upregulation of some of heat shock protein genes during fever conditions augments parasite infectivity [8]. We therefore enquired if the expression of PfHsp70-z is upregulated in *P*. *falciparum* parasites growing at the blood stage when they are subjected to heat stress. We observed that PfHsp70-z, like its cytosolic counterpart, PfHsp70-1 is upregulated in response to heat stress (Fig 2B and 2C). Furthermore, we validated the specificity of the α-PfHsp70-z antibodies by probing the parasite lysate using pre-immunisation serum which did not cross-react with PfHsp70-z (Fig 2A; *lower panel*).

### Secondary structure analysis of PfHsp70-z

**Fig 3:**
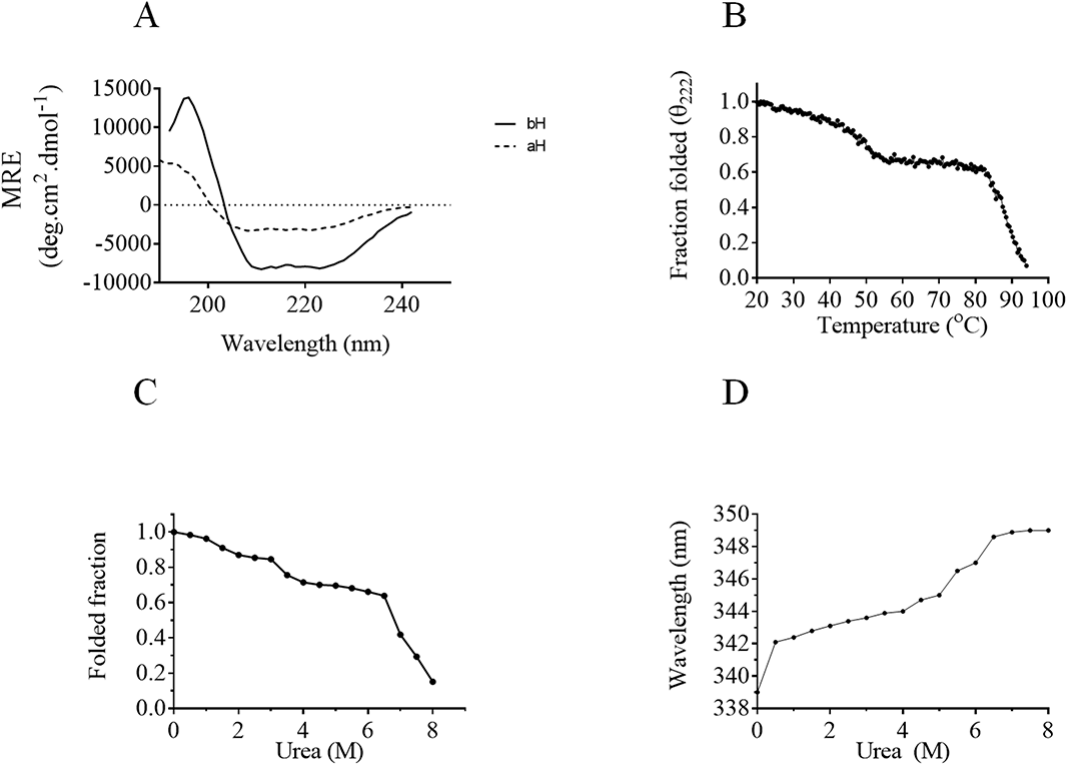
Analysis of the stability of PfHsp70-z to heat stress and urea treatment. Far UV spectra of recombinant PfHsp70-z protein before (bH) and after (aH) heat induced denaturation (A) The CD spectrum of PfHsp70-z was presented as molar residue ellipticity [MRE] (deg.cm^2^dmol^-1^). Assessment of the stability of PfHsp70-z to heat stress (B) and urea treatment (C). The stability of the protein was assessed by monitoring the folded status of the protein using CD spectroscopy (B,C); and tryptophan fluorescence (D).

To confirm the secondary structure of PfHsp70-z and to investigate the effect of heat stress on its stability, we conducted CD spectrophotometric analysis. To determine the secondary structure of PfHsp70-z incubated at 19°C e changes in CD ellipticity were recorded between wavelengths 190 nm and 260 nm (Fig 3A). The assay was repeated under varying temperature conditions (19°C– 95°C) (Fig 3A). The far-UV spectra exhibited a positive peak centred at 194 nm and two negative peaks at 209 and 221 nm, respectively, representing the helical character of the recombinant protein. The deconvolution of the CD spectra revealed that the full-length PfHsp70-z is predominantly composed of α-helices (62%), followed by β-strands (16%), β-turns (8%) and unordered (14%).

The stability of PfHsp70-z to heat stress was evaluated by assessing the folded state of the protein at 221 nm under temperature conditions (19°C– 95°C) (Fig 3B). At least 50% of the protein appeared stable at temperatures up to 80°C (Fig 3B). Furthermore, we assessed the stability of the protein to variable levels of urea (Fig 3C). We observed that more than 50% of the protein remained stable in the presence of up to 6.5 M urea.

In a separate experiment, the tertiary structure of PfHsp70-z was evaluated using tryptophan based fluorescence analysis of the protein in the presence of variable levels of urea (Fig 3D). We observed a red shift of emission with maximum peak at 350 nm corresponding to urea concentration of 6.5 M. This finding mirrors the observation we made by assessing the perturbation of the secondary structure of the protein in response to urea treatment (Fig 3C).

### PfHsp70-z is capable of self-association

**Fig 4:**
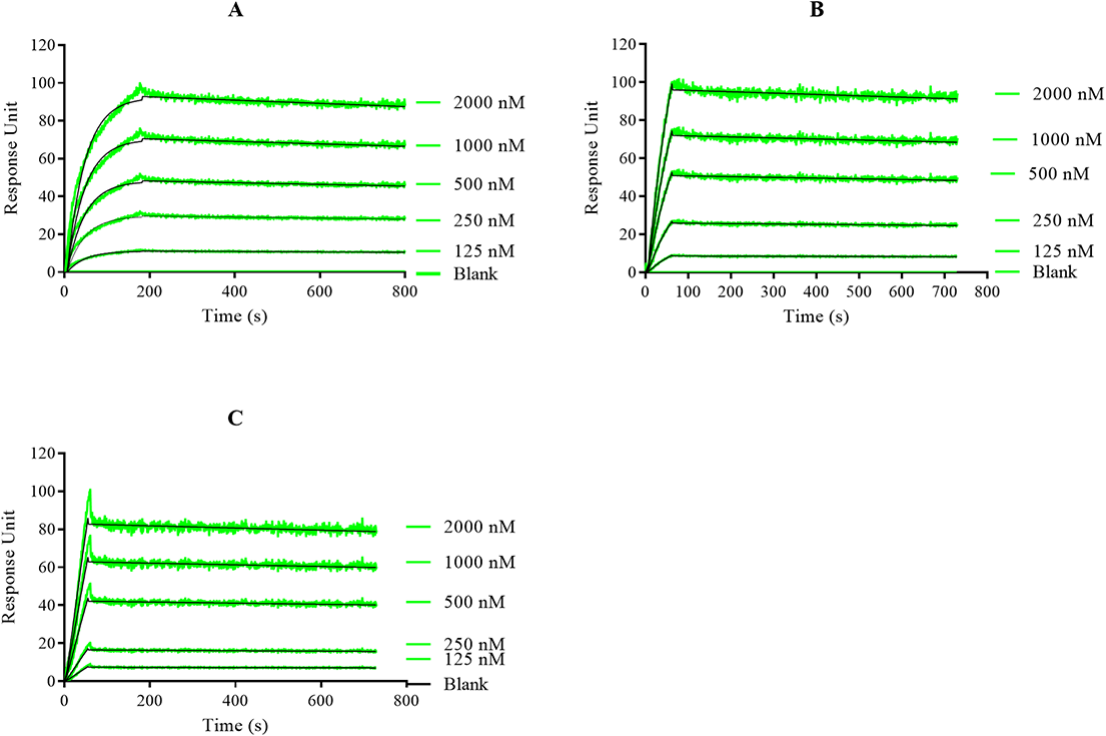
PfHsp70-z is capable of forming higher order oligomers. The SPR sensorgrams demonstrating concentration dependent association between PfHsp70-z mounted on the chip and PfHsp70-z introduced in solution. The assay was repeated in the absence of nucleotide (A); and in the presence of 5 mM ATP/ADP (B; C), respectively. Association was allowed for 2 min, and the dissociation was monitored for 10 min.

The purified recombinant PfHsp70-z appeared both as a 110 kDa monomer and a species of approximately 200 kDa based on SDS-PAGE and Western analyses (Fig 1B). We speculated that the band representing the species of approximately 200 kDa was that of a detergent resistant higher order of the protein, possibly a dimer. To further validate the oligomeric status of PfHsp70-z we conducted SPR analysis. The SPR data were analysed by applying the Langmuir kinetic model, to generate the *k*_*a*_, *k*_*d*_ and *K*_*D*_ values for the various SPR sensorgrams (Fig 4). We sought to analyse the ability of PfHsp70-z immobilised on the chip surface to interact with PfHsp70-z in solution. Based on the SPR analysis, PfHsp70-z (ligand) associated with its analyte form to generate higher order oligomers in a concentration dependent fashion (Fig 4). In addition, this association occurred in the absence of nucleotide as well as in the presence of either 5 mM ADP or ATP (Fig 4). The fact that this association occurs at nanomolar range (Table 1) suggests that the interaction is with fairly high affinity. To demonstrate that the self-association of PfHsp70-z was specific, BSA immobilised on the chip as a negative control was incapable of binding PfHsp70-z (Fig 4A–4C; lane labelled as ‘Blank’). Overall, our findings suggest that PfHsp70-z is capable of forming stable higher order oligomers.

**Table 1:** Kinetics data for the oligomerisation of PfHsp70-z.

Analyte	Presence of nucleotide	*k*_*a*_ (M^-1^s^-1^) [E+03]	*k*_*d*_ (s^-1^) [E-05]	*K*_*D*_ (nM)	Χ^2^	Reference
**PfHsp70-z**	-	12.0 (+/-0.3)	9.54 (+/-0.4)	7.97	3.06	This study
**PfHsp70-z**	5 mM ADP	6.62 (+/- 0.5)	7.60 (+/-0.5)	11.5	2.27	This study
**PfHsp70-z**	5 mM ATP	2.84 (+/-0.1)	3.20 (+/-0.1)	11.3	4.78	This study
**hHsp70**	-	ND	ND	4.5	ND	[38]

Table 1 legends: *k*_*a*_ association rate constant, *k*_*d*_ dissociation rate constant, *K*_*D*_ equilibrium constant, X^2^ correlation for SPR sensorgram fitting to the Langmuir model, ND not determined. Standard deviations of at least three independent protein batches are shown in parenthesis.

### PfHsp70-z is an ATPase

**Fig 5:**
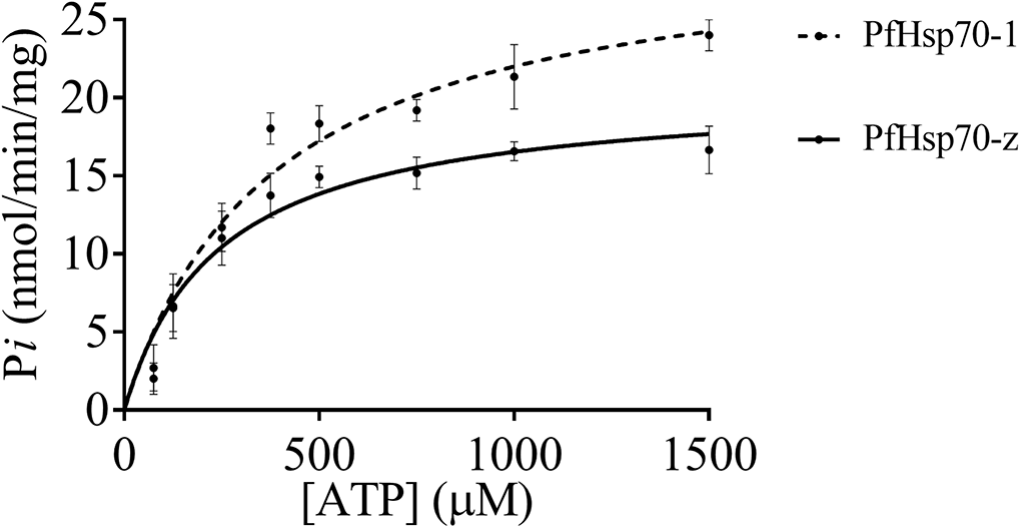
PfHsp70-z exhibits intrinsic ATPase activity. Analysis of the basal ATPase activities of PfHsp70-z and PfHsp70-1 and P*i* released was monitored at 595 nm using a direct colourimetric assay.

The basal ATPase activities of both PfHsp70-z and PfHsp70-1 were determined under variable initial ATP concentrations (up to 1000 μM). The kinetics for the generation of ADP was linear for all the assays that were conducted (Fig 5). The Michaelis-Menten plots were generated for at least three independent batches of PfHsp70-z and PfHsp70-1 (Fig 5). The turnover rate of PfHsp70-z we observed is comparable to that of PfHsp70-1 (Fig 5; Table 2); both of which lie within the same range as that reported for PfHsp70-1 based on a previous independent study [37]; (Table 1). PfHsp70-z exhibited a lower Km value (283 μM) compared to that of PfHsp70-1 (428 μM) suggesting that PfHsp70-z has higher affinity for ATP than PfHsp70-1. In addition, the catalytic efficiency (*K*_*cat*_/ *k*_*m*_) of PfHsp70-z was nearly two-fold higher than that of PfHsp70-1 (Table 1).

**Table 2:** A summary of the kinetics of the ATPase activities of PfHsp70-z and PfHsp70-1.

Protein	*V*_*max*_ (nmol/min/mg)	*K*_*m*_ (μM)	*K*_*cat*_ (mol^-1^)	*K*_*cat*_/*K*_*m*_	Reference
**PfHsp70-z**	19.8 (+/- 0.5)	283 (+/-3)	1.9	0.00671	This study
**PfHsp70-1**	24.6 (+/- 0.8)	428 (+/- 5)	1.6	0.00373	This study
**PfHsp70-1**	14.6	616.5	1.03	0.00167	[37]

Table 2 legends: Values for the *V*_*max*_, *K*_*m*_, and *k*_*cat*_ were determined as shown. Standard deviations shown represent at least three independent assessment made using separate protein batches.

## Discussion

PfHsp70-z is an essential molecule which has been reported to occur in parasite cytosol and is thought to inhibit the aggregation of malarial asparagine repeat rich proteins [25]. The protein is thus thought to mask the effects of the stress that the parasite encounters in the host. Furthermore, PfHsp70-z and PfHsp70-1 are expressed at the clinical phase of malaria and their expression is reported to mirror malaria pathology [8]. To the best of our knowledge, this is the first study to report the over-expression, purification and in vitro biochemical characterisation of PfHsp70-z. Chloroquine resistance in *P*. *falciparum* is mapped to a 36 kb segment of Chromosome 7, which includes the *PfHsp70-z* (*PfCg4)* gene [27]. Therefore, the characterisation of PfHsp70-z towards understanding its broad functions is important. For example, Hsp110-Hsp70 complexes have been implicated in cell division where they modulate the activity of the molecules kenesin-5 motor and cin8, which are required for spindle elongation in yeast and mammalian cells [28]. The modulation of spindle elongation by molecular chaperones might be a mechanism in which cell division may be controlled under proteostatic stress. The rapid growth cycles of *P*. *falciparum* in the host facilitates its survival and may influence its pathogenesis [29]. In light of the possible broad spectrum of functions for this protein its characterisation is important.

Firstly, we observed that PfHPfHsp70-z is induced by heat stress in malaria parasites cultured at the blood stage (Fig 2A). Its cytosolic homologue, PfHsp70-1 is known to be also stress-inducible [7]. This suggests that these two proteins may cooperate in maintaining protein quality in the parasites. Indeed, sequence data suggest that PfHsp70-z is a possible nucleotide exchange factor of PfHsp70-1 [24]. We further sought to establish the secondary structure of PfHsp70-z. The protein possesses α-helix and β-sheets estimated to be 50%- 62%%; and 10%- 16% (Fig 3A), respectively, which correlates with the reported structural constitution of its yeast Hsp110 homologue, Sse1 [39]. PfHsp70-z also possesses β-turns (8%) and a fairly significant amount of unordered segments (14%). Our findings suggest that at least 50% of PfHsp70-z is heat stable at temperatures up to 80°C (Fig 3B). The resilience of PfHsp70-z to heat stress is consistent with its role as a molecular chaperone [25] as the functions of molecular chaperones is particularly important in response to the development of cellular stress.

The recombinant PfHsp70-z protein that we purified appeared both as a monomer and possibly as a dimer (Fig 1B). We conducted SPR analysis to validate that of PfHsp70-z molecules are capable of self-association (Figs 1 and 4). The formation of PfHsp70-z higher order oligomers of PfHsp70-z is nucleotide-independent and the interaction is associated with fairly high affinity (Fig 4; Table 1). Our findings are in agreement with independent studies that have suggested that canonical Hsp70 and Hsp110 homologues are capable of forming dimers [38][40].

We also established that PfHsp70-z is an ATPase (Fig 5). The protein demonstrated higher catalytic efficiency than PfHsp70-1 (Table 2). It has been reported previously that PfHsp70-z provides cytoprotection to malaria parasites by preventing the aggregation of asparagine repeats rich proteins of *P*. *falciparum* [25]. Taking into account that about 24% of *P*. *falciparum* proteins possess asparagine rich repeats, it is not surprising that this protein is essential [41]. Hsp110s are largely thought to act as holdases that are capable of holding client proteins in a folding competent manner under thermal stress [18]. They are thought to subsequently pass their substrates to other chaperones such as canonical Hsp70s that are capable of refolding proteins. It is for this reason that their ATPase subdomain is thought to be dispensable as the holdase function of Hsp70/Hsp110 chaperones is independent of ATP hydrolysis [16][18]. On the other hand, some studies have suggested that ATP binding may be important for their holdase function, and not necessarily ATP hydrolysis [42][43]. Although it remains to be established whether nucleotide binding influences the independent chaperone activity of PfHsp70-z, the current findings suggest that it is capable of hydrolysing ATP. Apart from their role as holdase chaperones, Hsp110s are some of the most prominent NEFs for the canonical Hsp70 partners [15]. Since PfHsp70-z and PfHsp70-1 are both stress induced and co-localize in the parasite cytosol, it is highly likely that PfHsp70-z is a nucleotide exchange factor of PfHsp70-1.

Our study demonstrates direct evidence suggesting that PfHsp70-z is an ATPase that is capable of forming higher order oligomers. The protein is heat stable and its expression is induced by heat stress suggesting an important role of this protein in the development of malaria parasites. Optimization of the over-production of purified, functional recombinant PfHsp70-z paves way for further biochemical analysis to establish its functions in the development of malaria parasites.
